# Stem cells, cell therapies, and bioengineering in lung biology and disease 2021

**DOI:** 10.1152/ajplung.00113.2022

**Published:** 2022-06-28

**Authors:** Laertis Ikonomou, Mattias Magnusson, Ruben Dries, Erica L. Herzog, Robert E. Hynds, Zea Borok, Jin-Ah Park, Steven Skolasinski, Janette K. Burgess, Leigh Turner, Sarah M. Mojarad, John E. Mahoney, Thomas Lynch, Mareike Lehmann, Victor J. Thannickal, Jamie L. Hook, Andrew E. Vaughan, Evan T. Hoffman, Daniel J. Weiss, Amy L. Ryan

**Affiliations:** ^1^Department of Oral Biology, University at Buffalo, State University of New York, Buffalo, New York; ^2^Division of Pulmonary, Critical Care and Sleep Medicine, Department of Medicine, University at Buffalo, State University of New York, Buffalo, New York; ^3^Division of Molecular Medicine and Gene Therapy, Lund Stem Cell Center, Lund University, Lund, Sweden; ^4^Section of Hematology and Medical Oncology, Department of Medicine, Boston University School of Medicine, Boston, Massachusetts; ^5^Yale Interstitial Lung Disease Center of Excellence, Pulmonary and Critical Care Medicine, Yale School of Medicine, New Haven, Connecticut; ^6^Epithelial Cell Biology in ENT Research Group, Developmental Biology and Cancer Department, UCL Great Ormond Street Institute of Child Health, University College London, London, United Kingdom; ^7^Division of Pulmonary, Critical Care and Sleep Medicine, University of California, San Diego, California; ^8^Department of Environmental Health, Harvard T.H. Chan School of Public Health, Boston, Massachusetts; ^9^Department of Medicine, University of Minnesota, Minneapolis, Minnesota; ^10^Department of Pathology and Medical Biology, Groningen Research Institute for Asthma and COPD, University of Groningen, University Medical Center Groningen, Groningen, The Netherlands; ^11^Department of Health, Society, and Behavior, University of California, Irvine Program In Public Health, Irvine, California; ^12^Engineering in Society Program, Viterbi School of Engineering, University of Southern California, Los Angeles, California; ^13^Cystic Fibrosis Foundation, Lexington, Massachusetts; ^14^Department of Surgery, Carver College of Medicine, University of Iowa, Iowa City, Iowa; ^15^Institute of Lung Health and Immunity, Comprehensive Pneumology Center Munich, Helmholtz Zentrum München, Munich, Germany; ^16^John W. Deming Department of Medicine, Tulane University School of Medicine, New Orleans, Louisiana; ^17^Division of Pulmonary, Critical Care, and Sleep Medicine, Department of Medicine, Icahn School of Medicine at Mount Sinai, New York City, New York; ^18^Global Health and Emerging Pathogens Institute, Department of Microbiology, Icahn School of Medicine at Mount Sinai, New York City, New York; ^19^Department of Biomedical Sciences, School of Veterinary Medicine, University of Pennsylvania, Philadelphia, Pennsylvania; ^20^Department of Medicine, University of Vermont, Burlington, Vermont; ^21^Hastings Center for Pulmonary Research, Department of Medicine, University of Southern California, Los Angeles, California; ^22^Department of Stem Cell and Regenerative Medicine, University of Southern California, Los Angeles, California; ^23^Department of Anatomy and Cell Biology, Carver College of Medicine, University of Iowa, Iowa City, Iowa

**Keywords:** bioengineering, differentiation, extracellular matrix, lung regeneration, pluripotent stem cells

## Abstract

The 9th biennial conference titled “Stem Cells, Cell Therapies, and Bioengineering in Lung Biology and Diseases” was hosted virtually, due to the ongoing COVID-19 pandemic, in collaboration with the University of Vermont Larner College of Medicine, the National Heart, Lung, and Blood Institute, the Alpha-1 Foundation, the Cystic Fibrosis Foundation, and the International Society for Cell & Gene Therapy. The event was held from July 12th through 15th, 2021 with a pre-conference workshop held on July 9th. As in previous years, the objectives remained to review and discuss the status of active research areas involving stem cells (SCs), cellular therapeutics, and bioengineering as they relate to the human lung. Topics included *1*) technological advancements in the in situ analysis of lung tissues, *2*) new insights into stem cell signaling and plasticity in lung remodeling and regeneration, *3*) the impact of extracellular matrix in stem cell regulation and airway engineering in lung regeneration, *4*) differentiating and delivering stem cell therapeutics to the lung, *5*) regeneration in response to viral infection, and *6*) ethical development of cell-based treatments for lung diseases. This selection of topics represents some of the most dynamic and current research areas in lung biology. The virtual workshop included active discussion on state-of-the-art methods relating to the core features of the 2021 conference, including in situ proteomics, lung-on-chip, induced pluripotent stem cell (iPSC)-airway differentiation, and light sheet microscopy. The conference concluded with an open discussion to suggest funding priorities and recommendations for future research directions in basic and translational lung biology.

## Graphical abstract

**Figure:**
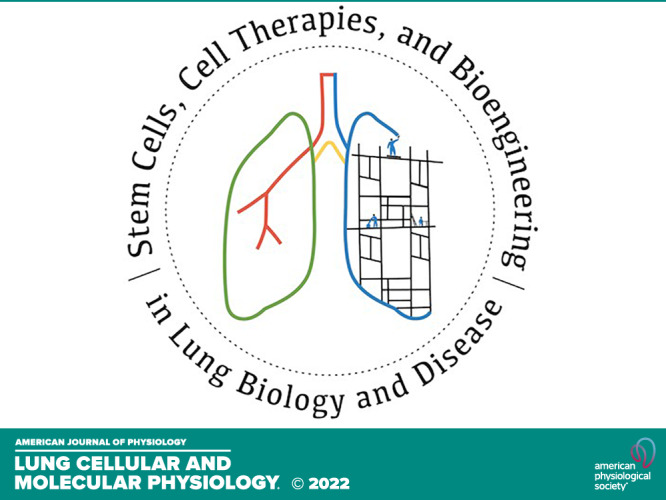

## INTRODUCTION

The 9th biennial conference followed in the footsteps of the previous eight conferences providing an active and current discussion of recent advances in the field of lung stem cells (SCs), cellular therapies, and bioengineering. For 2021, Dr. Amy L. Ryan [University of Southern California (USC), currently at the University of Iowa] took over as Chair supported by Vice-Chair Dr. Laertis Ikonomou (University at Buffalo) and Emeritus Chairs Dr. Daniel Weiss (University of Vermont) and Dr. Darcy Wagner (Lund University). The application of new and emerging technologies to advance basic and translational lung biology remained a core theme across the scientific sessions that all centered around futhering our understanding of the cellular, molecular, and biomechanical regulation of lung regeneration. The scientific sessions and discussion centered around core issues hampering progress in lung regeneration. These issues included *1*) identification and function of lung stem cell populations; *2*) understanding how different niche factors regulate lung stem cells in the context of regeneration and lung function; *3*) developing more complex, tissue level models of the lung to understand regulation by mechanical factors such as stretch and compression; and *4*) delivery and functional engraftment of cells into the regenerating lung.

Building on prior conferences, the regulation of airway stem cells by their immediate micorenvironment including interactions with immune cells and extracellular matrix (ECM) was heavily discussed. Such interactions were also put in the context of airway regeneration where ECM biomechanics, aging, and viral infection were all examined in the setting of driving innovation in regenerative strategies for acute and chronic lung diseases. New single-cell omics technologies are being developed at an unprecedented pace. This is especially true within the emerging field of spatial omics (1–3), which allows for the analysis of single cells within the intact tissue. This new type of information is crucial for a deeper understanding of the effects of spatial organization and cell-cell and cell-matrix cross talk on gene expression. Not surprisingly, spatial transcriptomics was voted as the “method of the year 2020,” by *Nature Methods* (4). Challenges in the in vitro expansion of airway epithelial cells was a theme transcending many talks as the pursuit of functional tissue regeneration remains a long-term goal for many laboratories studying human lung diseases. As our understanding of cellular heterogeneity expands, we are becoming increasingly aware of the role of cell-cell and cell-matrix interactions or cross talk between epithelial cells and resident or circulating immune cells or mesenchymal cells play in determining fate decisions and functional changes in the lungs. To make continuing progress toward the development of safe and efficacious cellular therapeutics, comprehensive understanding of these processes will be critical to determine the long-term consequences of cellular therapy from endogenous or exogenous cellular sources.

Due to the ongoing COVID-19 pandemic, the popular “hands-on” pre-conference workshop was converted to an interactive online format. Workshops comprised of pre-recorded training were made available to all attendees one week before a live question-and-answer session on the pre-conference workshop day. This format, while missing some of the features of the pre-pandemic in-person format, did allow for increased time to be dedicated to each session and attendees could participate in all sessions rather than selecting three sessions as in previous years. The workshop featured previous core trainings including decellularization of whole lungs, preparation of precision cut lung slices, and ex vivo culture techniques. In addition, new sessions on induced pluripotent stem cells (iPSCs), light sheet microscopy, in situ proteomics, and lung-on-chip reviewed some of the latest technological advances impacting the field of lung diseases and airway regeneration.

The 2021 conference concluded with a highly interactive question and discussion session addressing key themes from the conference. This report summarizes the topics featured throughout the conference, highlighting key areas for continued innovation and funding to enable the field to sucessfully advance. The full pre-conference and conference program are available from Figshare at https://doi.org/10.6084/m9.figshare.19406570.

## SESSION I: TECHNOLOGICAL ADVANCES FOR IN SITU ANALYSIS OF TISSUES

By further extending their newly developed microfluidic-based spatial omics technology, DBiT-seq (Deterministic Barcode in Tissue) (5), Dr. Yang Liu (Yale University), revealed that their platform can now be used on formalin-fixed, paraffin-embedded tissues (FFPE) with the capacity to detect as many as 300 proteins at once. DBiT-seq technology extends beyond gene expression and can include spatial epigenetics using both ChIP-seq (chromatin immunoprecipitation) and ATAC-seq (assay for transposase-accessible chromatin) chemistry. Importantly, DBiT-seq is a user friendly and affordable spatial multi-OMICS platform that will likely become widely used in the study of lung biology.

Single-cell heterogeneity can potentially be explained by transcriptional bursts caused by the diverse activation kinetics of various transcription factors (TFs) (6). Similar mechanisms have been shown to play a key role in drug resistance in cancer (7); though the underlying mechanisms are not yet well understood. Using live-cell particle imaging, Dr. Timothee Lionnet (New York University Langone Medical Center) has developed methods to measure variability in real-time gene expression using microscopy. They found that TFs exhibit a wide range of functional variability, and that the bursting kinetics are independent of how long the TF binds to chromatin and instead dependent on transcription initiation and chromatin remodeling. This system provides a toolbox that can be particularly useful for developing synthetic activators to reprogram cells.

In addition to transcriptional regulation, lung cells such as alveolar epithelial type 1 (AT1) cells undergo major morphological changes during development (8). Studying morphogenesis in vivo is particularly challenging with flat cells such as AT1 cells. Graduate student, Vera Hutchison (MD Anderson Cancer Center) has developed new reporter mice in which it is possible to detect six different subcellular structures within a single cell. Using this model, she revealed that AT1 cells display prominent lysosomes and remarkable mitochondrial distribution in vivo. Continued evolution of this technology will likely enhance our ability to study lung cell biology in vivo.

Taken together, these novel technologies have the promise to substantially improve our understanding of the regulatory mechanisms during lung homeostasis and diseases. These technologies are, however, still in their infancy and several challenges remain. For example, the resolution of spatial proteomics needs improving to be able to capture the whole proteome at a single-cell level. In addition, understanding how the noncoding genome contributes to transcriptional regulation is not addressed. Finally, the generation of huge data sets (from both omics and imaging) reveal the importance of developing affordable data storage and computing power solutions. Acknowledging these challenges, we anticipate that the development of novel, more precise, and efficient technologies for evaluting single-cell biology will continue to accelerate.

## SESSION II: THERAPEUTIC IMMUNOMODULATION OF THE LUNG

Pulmonary applications of cell-based therapies have expanded to encompass immunomodulation by a widening range of cell types. For example, as shown by Dr. Ke Cheng (NC State University and UNC-Chapel Hill), administration by inhalation of cells isolated from lung spheroids, prepared from surgical and bronchoscopic lung biopsies (9, 10), stimulates alveolar regeneration in a bleomycin model of pulmonary fibrosis (9). The exosome and nonexosome components of the secretome from these cells recapitulate these findings via mechanisms involving canonical transforming growth factor-β1 (TGF-β1) signaling and monocyte chemotractant protein 1 (MCP-1) expression (11). Furthermore, cell-mimicking nanodecoys, engineered to neutralize SARS-CoV-2 (severe acute respiratory syndrome coronavirus 2), mitigate viral replication and inflammatory lung injury in nonhuman primate models of COVID-19 (coronovirus disease 2019) (12). Identification of exosome cargo and additional study of nanodecoys are exciting areas for future investigation.

Treatment of pulmonary alveolar proteinosis (PAP) by pulmonary macrophage transfer (PMT) has been achieved in mouse models (13). Dr. Bruce Trapnell (University of Cincinnati) has demonstrated that transfer of *GM-CSF* gene-corrected macrophages derived from autologous iPSCs (14) or hematopoietic stem cells (HSCs) (13) improves both pulmonary and systemic manifestations of disease and elucidates the critical role of GM-CSF in the feedback regulation of the alveolar macrophage population size. Gene-corrected PMT is, therefore, a durable and efficacious therapy that can be achieved without myeloablation or immunosuppression and clinical trials are scheduled to start soon. New areas of study related to this work include the importance of macrophage quorum sensing, relative contributions of interstitial versus alveolar macrophages, and PMT’s therapeutic potential in other conditions.

The critical role of macrophages in lung homeostasis necessitates the need for well-defined phenotypic metrics. Dr. Patrick Hume and colleagues (National Jewish Health) augment the classic methods of macrophage surface marker expression, transcriptome, and function by quantifying location. Using design-based stereology, a systematic approach employing three-dimensional (3-D) reconstruction of randomly selected regions of the right upper lobe of lungs obtained from humans who had died of nonpulmonary causes, identified CD206^+^CD43^+^ interstitial macrophages located primarily in the alveolar septa, compared with airways and perivascular spaces, and also exceeded quantities of alveolar macrophages (15). The lungs of smokers without clinically notable lung disease contained a significant increase in interstitial, but not alveolar, macrophages (15). These findings open numerous new areas of exploration including evaluating the feasibility of this method, generalizability across all lung zones, relationship to prior studies that yielded qualitative and quantitative differences, and of course alterations in disease.

Tissue sampling limitations may be circumvented by isolating the epithelial lining fluid via mini-bronchoalveolar lavage (BAL). Dr. Katherine Wick and colleagues (University of California, San Francisco) performed secondary analysis of the “Human Mesenchymal Stromal Cells For Acute Respiratory Distress Syndrome” (START) trial (ClinicalTrials.gov Identifier: NCT02097641), a Phase 2a safety study in which patients with acute respiratory distress syndrome (ARDS) were randomized to receive peripheral infusion of mesenchymal stromal cells (MSCs) or vehicle (16). This work found that mini-BAL measurements, but not plasma measurements of total protein for epithelial barrier function, angiotensin 2 (ANG2) levels for endothelial function, and interleukin-6 (IL-6) or tumor necrosis factor receptor 1 (TNFR-1) for inflammation were attenuated by MSC infusion. ANG2 concentrations were independently associated with fewer days alive without the need for mechanical ventilation whereas receptor for advanced glycation end products (RAGE) was independently associated with radiographic measures of pulmonary edema (17). Although additional work is needed to validate the outcome predictive properties of these mediators, mini-BAL appears to be safe, feasible, and potentially superior to commonly used surrogates in the peripheral blood.

## SESSION III: NEW INSIGHTS INTO CELLULAR PLASTICITY IN THE LUNG

The lineage relationships between epithelial cell types within the lung of both mice and humans are being revealed in ever more detail. During homeostasis, the tracheal, bronchiolar, and alveolar stem cell compartments are maintained by local progenitor cell populations (18). Basal cells give rise to mucosecretory and multiciliated populations, as well as less frequent cell types such as pulmonary neuroendocrine cells (PNEC) and ionocytes in the cartilaginous airways. Club cells can give rise to both mucosecretory and multiciliated lineages in the bronchiolar epithelium, whereas alveolar epithelial type II (AT2) cells produce AT1 cells in the alveolus. In recent years, we have begun to appreciate that these cell fate trajectories are more flexible during regenerative responses. Bronchioalveolar stem cells (BASC) are found at bronchioalveolar duct junctions (19) and can give rise to bronchiolar or alveolar lineages in vivo (20), whereas club cells have the capacity to give rise to basal cells after severe basal cell depletion (21). AT1 cells are also capable of contributing to alveolar regeneration during neonatal injury in mice (22), although adult AT1 cells do not retain this same functionality (22), suggesting that age is an important factor in lung plasticity.

Dr. Carla Kim (Boston Children’s Hospital) presented data on two studies in which they have used organoid models to study lung disease. To evaluate the early stages of lung cancer initiation, the team has combined mouse models of oncogenic *Kras G12D* and loss of *Trp53* with organoid culture models (23, 24). After isolation from *Kras G12D* mice, AT2 cells were infected with adeno-Cre and plated in organoid culture conditions. Histological analyses show tumor morphology coincident with enlarged, pleomorphic nuclei and simultaneous deletion of *Trp53* induces indicators of aggressive tumor behavior, such as giant multinucleated cells, consistent with data from mouse models (25, 26). Tumor organoids formed lung tumors in vivo following transplantation. RNA sequencing (RNAseq) and immunofluorescence analyses showed a loss of AT2 cell markers, such as lysozyme 2 (*Lyz2*) and surfactant protein C (*Sftpc*), and gain of developmental markers, such as SRY-box transcription factor 9 (*Sox9*) and high mobility group A2 (*Hmga2*), following induction of *Kras*. These data were confirmed in single-cell RNAseq (scRNAseq), where substantial differences between control and *Kras G12D*-activated AT2 cells were observed. Data from human patients and iPSCs are consistent with the loss of AT2 cell-associated genes being an early event following *KRAS* mutation in lung adenocarcinoma.

Transplantation models are an important tool to study the potential of stem cells within an in vivo environment. To investigate the potential of lung organoids to engraft and regenerate the airways in the bleomycin model of lung injury, Dr. Kim demonstrated that Sca1-negative, AT2 cell-derived organoids were administered and found to engraft and express AT2 markers over two weeks posttransplantation. scRNAseq showed that transplanted cells could be identified that have transcriptional profiles similar to those of both native AT2 cells and primed AT2 cells, whereas functional analyses suggests that cell transplantation partially ameliorated fibrosis. The transplanted cells retain organoid-formation capacity and proliferate to a similar extent after secondary bleomycin injury. Sca1^+^ multipotent organoids also generated both alveolar and airway lineages in vivo. Further development of these lung injury transplantation models will provide new insights into lung regeneration and provide a model to study human and/or genetically modified stem cells.

Claudins are structural components and regulators of tight junctions, and thus play a key role in epithelial permeability. Dr. Zea Borok (University of California, San Diego) presented data addressing the role of claudin-18 (*Cldn18*) in lung regeneration and cancer. CLDN18 is expressed in alveolar epithelial cells and is required for normal tight junction structure and function. *Cldn18^−^*^/−^ mice had no overt respiratory phenotype, but displayed increased lung solute permeability, increased fluid clearance, and actin cytoskeleton changes (27). Surprisingly, *Cldn18*^−/−^ mice were protected from pathological changes associated with bleomycin lung injury, such as increased BAL protein, decreased compliance, and lung fibrosis. Furthermore, *Cldn18*^−/−^ mice display organ enlargement, including an approximately sixfold increase in parenchymal volume and increased cellularity as a result of increased AT2 cell proliferation in the lung (28). Mechanistically, CLDN18 interacts with yes associated protein (YAP) and large tumor suppressor kinase 1 (LATS) family proteins and the loss of CLDN18 leads to increased nuclear localization of p-YAP. Multiple possible mechanisms, including the hypotheses that CLDN18 might spatially coordinate YAP-LATS interactions at tight junctions or that cytoskeletal changes cause Rho-induced YAP activation, require further interrogation. Interestingly, *Cldn18*^−/−^ mice have a high incidence of lung adenocarcinoma in old age (29) and overexpression of Cldn18 suppresses xenograft tumor growth in vivo, suggesting a role in lung tumorigenesis. In this case, Cldn18 overexpression inhibits insulin growth factor 1 (IGF-1) and YAP/TAZ signaling to reduce p-AKT signaling and suppresses tumor progression (29). Impressive progress in the use of both in vitro and in vivo models to interrogate the molecular basis of lung cellular plasticity was highlighted in this session. We were also reminded of the challenges relating to differences between mouse and human lung biology, protocol differences between investigators, and the difficulty of preconditioning for cell engraftment in a translational setting.

## SESSION IV: STEM CELL SIGNALING IN LUNG REMODELING

The complex interactions between stem cells and the niches in which they reside in both normal and diseased lung are increasingly being elucidated as are the cell signaling pathways involved in matrix effects on stem cell homeostasis. This includes growing understanding of basal airway epithelial cells and their potential role in malignant transformation. Dr. Tien Peng (University of California, San Francisco) presented data underlying a core hypothesis that metaplasia represents abnormal repair. This can result in development of fibrosis and/or malignant transformation with appearance of aberrant ectopic basal cells. Mouse model data suggest a role of glioma associated protein (Gli1)-mediated hedgehog signaling resulting in bone morphogenic protein (BMP) antagonism and metaplastic basal cell differentiation. Restoration of BMP signaling attenuates metaplastic changes and demonstrates both mechanistic pathways and means by which interventions may be made. However, the comparative normal and aberrant differentiation pathways between mouse and human lineages are still being elucidated, particularly with respect to differentiation of AT2s to AT1s and the roles of the surrounding niche environment.

There is also further growing appreciation of the complexity of AT2s and intermediate transitional states during AT2 to AT1 cell differentiation, both as part of normal repair after injury or aberrant repair that leads to pathologic states notably fibrosis. Dr. Jonathan Kropski (Vanderbilt University) reviewed scRNAseq approaches utilized by a growing number of groups to characterize growing appreciation of epithelial cell and also fibroblast diversity in fibrotic lungs. These approaches have also been recently utilized to characterize aberrant epithelial cell repair pathways in SARS-CoV-2-infected lungs and have identified some striking similarities, as well as differences compared with epithelial repair pathways in idiopathic pulmonary fibrosis (IPF) lungs. Using a repetitive bleomycin injury model in mice, respective complementary roles in repair were identified for cytokeratin 5 (Krt5)+ basal airway epithelial, Krt19+ transitional, and Scgb1a1+ progenitor cells. Sophisticated mRNA splicing trajectory methodologies provide further insight in the respective roles of different cell types in distinct injury and repair circumstances.

In addition to evolving cell and molecular biological analytical techniques, advances in imaging approaches have continued to evolve and to provide novel information on lung progenitor cell behaviors. Hani Alsafadi, graduate student in the Lung Bioengineering and Regeneration Laboratory at Lund University, presented data on the power of light sheet fluorescence microscopy to delineate and isolate different progenitor populations. Coupled with advanced approaches to perfusion-based whole lung immunolabeling, this results in improved quality of staining and allows for high-resolution imaging. There are multiple potential applications of this and other evolving labeling and imaging approaches.

## SESSION V AND VI: CAREERS IN STEM CELLS, CELL THERAPIES, AND LUNG BIOENGINEERING

Two sessions focused on career development were held including a keynote talk on the pursuit of translational biomedical science in academia by Dr. Gordana Vunjak-Novakovic (Columbia University), with a panel discussion focused on women and diversity and concurrent panel discussion sessions on finding a position/starting your own laboratory and funding and financial management of a laboratory. Further details on these sessions are available at https://doi.org/10.6084/m9.figshare.19406570.

## SESSION VII: MATRIX BIOMECHANICS AND STEM CELL DYNAMICS IN LUNG HEALTH AND DISEASE

During embryonic development and in maintenance of lung homeostasis, epithelial and mesenchymal interactions are critical (30, 31), with interactions being sequential or reciprocal depending on the context. Under disease conditions, communication between the epithelium and the surrounding mesenchyme could determine pathologic processes. In session VII, focus was on advancing knowledge in understanding cellular mechanisms of dysregulated lung epithelial cells in IPF and emerging therapeutic approaches, including stem cell-based therapies and engineered lung transplantation.

In diffuse parenchymal diseases, including IPF, one of the central pathologic features is dysfunction of lung epithelial cells, including AT2 cells. Dr. Michael Beers (University of Pennsylvania) discussed the cellular mechanisms by which AT2 cells undergo pathologic transitions toward fibrotic epithelial endophenotypes. As both progenitor cells and biological regulators, homeostatic functions of AT2 cells are maintained by elegant cellular quality control (QC) such as ubiquitin-proteasome system, unfolded protein response (UPR), macroautophagy, mitophagy, telomere maintenance, and metabolism, in response to various internal and external insults (32). However, sustained dysfunctions of these QC components cause the acquisition of cellular endophenotypes that contribute to the production of fibrotic mediators and aberrant repair, as observed in IPF. Understanding mechanisms of the disrupted QC in aberrant cellular endotypes of AT2 cells can reveal new targets and therapeutic strategies for developing treatments for IPF.

In patients with IPF, an emerging therapeutic option is a replacement of the impaired lung with an engineered lung (33, 34). To engineer lungs, which could safely replace terminally diseased lungs, a comprehensive understanding of epithelial and mesenchyme interactions is required. The group led by Dr. Sarah Gilpin (United Therapeutics) uses an ultrahigh-resolution 3-D bioprinting technology to assemble organ scaffolds, which serve as a platform. Subsequently, the quality of these platforms can be determined by evaluating the cellularization processes, including attachment of the implemented epithelial cells to the scaffold, proliferation and differentiation of the epithelial cells, with eventual emergence of lung-like phenotype and functions. In functional studies at the early stage, the engineered mini lobes exhibit the capacity of gas exchange, which is one of the most critical functions of the lung. Beyond this promising progress, further studies will be required for recapitulating the homeostasis and normal injury responses in the engineered lung.

One of the potential therapeutic options for IPF that have long been considered is a cell-based therapy using lung resident MSCs (L-MSCs). However, the multifaceted roles of L-MSCs in multiple biological processes also raise safety concerns (35, 36). The concerns about the use of MSCs are mostly due to a lack of mechanistic understanding of L-MSCs in IPF. To advance our knowledge about the physiological functions of L-MSCs, Dr. Aina Martin-Medina (Balearic Islands Health Research Institute) discussed the role of L-MSCs in the alteration of the lung microenvironment. In L-MSCs isolated from patients with IPF, mitochondrial morphology and functions are dysregulated. In L-MSCs cultured from donors with both non-IPF and IPF, transforming growth factor beta (TGF-β1) induces expression of profibrotic mediators, plasminogen activator inhibitor 1 (PAI-1) and collagen type 1 alpha 1 (COL1A1). The degree of increased expression of both mediators was greater in L-MSCs from non-IPF, suggesting that PAI-1 and COL1A1 in L-MSC from patients with IPF were already elevated at baseline.

In summarizing session VII, cellular dysfunction resulting from the disruption of cellular QC mechanisms in IPF and the potential therapeutic options for IPF, including L-MSCs and engineered lungs were all discussed.

## SESSION VIII: ENGINEERING MODELS OF LUNG DISEASE AND REGENERATION

An essential requirement for understanding lung diseases and developing therapies to treat them is an effective biological model. The traditional method of growing single layers of homogeneous cells on plastic culture dishes, while yielding valuable information to date, is now recognized for its limitations. Consequently, substantial research is currently focused on developing more complex models for informing the development and treatment of lung disease. These alternatives were explored in the Dame Julia Polak Memorial Bioengineering session, “Engineering Models of Lung Disease and Regeneration”.

In diseases such as IPF and pulmonary hypertension (PH), the fibrotic changes stiffen the lung matrix, which in turn induces fibrosis in neighboring tissue resulting in spread of the pathologic phenotype. To better understand the temporal development of this process, Dr. Chelsea Magin and colleagues (University of Colorado) developed a system utilizing decellularized pulmonary extracellular matrix (DECM) within which the initial soft scaffold can be selectively stiffened in both a spatially and temporally specific manner by incorporating photo-crosslinkable compounds. They found that epithelial cells developed a fibrotic phenotype (expressing increased alpha smooth muscle actin (α SMA) and COL1A1) when the matrix is stiffened. This DECM culture system can be produced from healthy or diseased lung ECM potentially providing a tool for understanding the interplay between stiffness and biochemical composition in the development of fibrosis.

In addition to cell-matrix interactions, cell-cell interactions are also important when modeling in vivo systems. Neonatal hyperoxia is well known to induce bronchopulmonary dysplasia (BPD) and it is presumed that AT2 cells are central to this process. To explore BPD progression ex vivo, Dr. Jennifer Sucre and colleagues (Vanderbilt University Medical Center) developed a 3-D organotypic culture system where growing AT2 cells in a complex collagen/Matrigel matrix in contact with fibroblasts created an environment that supported their longitudinal study. Studying the effects of hyperoxia on AT2 cells over time revealed increased β-catenin and WNT5a signaling, which mirrors elevated WNT5a signaling in lung tissue from infants with BPD, and led to the discovery that a WNT5a inhibitor abrogates some of the effects of hyperoxia in precision cut lung slices.

Dr. Janna Nawroth and colleagues (Ryan Laboratory, University of Southern California) used a lung-on-a-chip system with an endothelialized vascular compartment containing flowing media separated by a membrane from an epithelialized airway compartment that can be cyclically stretched. They demonstrated the utility of the system for drug development by showing reduced neutrophil transmigration from the vascular compartment into the airway compartment, in the context of an asthma model, following treatment with an agent that blocks neutrophil chemotaxis. Lung-on-chip also facilitates investigation of the influence of the mechanical microenvironment on cell development. The airway matrix can be exposed to airflow relevant shear stresses, accompanied by cyclical strain associated with breathing. Cells differentiated on the lung-on-chip develop a phenotype more reflective of small airways when compared with traditional transwell cultures.

The potential importance of innovative cell culture techniques was further highlighted in Dr. Chandani Sen and colleagues (University of California, Los Angeles) investigations in small cell lung cancer (SCLC), a malignancy with a particularly high relapse rate. Three-dimensional alveolar organoids were formed using cell-coated degradable beads, with subsequent dissipation leaving an alveolar-like structure. Compared with two-dimensional (2-D) culture, the 3-D tumor organoid cultures were closer to native tumors in histologic appearance and neuroendocrine hormone expression, while recapitulating cancer relapse after chemotherapy. The scalability of this system elevates its promise as a tool for high-throughput drug screening during new drug development or to aid in selecting patient-specific therapies using allogenic tumor organoids.

## SESSION IX: DIRECT-TO-CONSUMER CELL-BASED INTERVENTIONS FOR RESPIRATORY DISEASES: STRATAGEMS AND COUNTERMEASURES

Acknowledging the promise of stem cell research and regenerative medicine, thousands of clinics take advantage of individuals with serious health problems by advertising cell-based interventions that have not been approved by regulatory bodies, do not fall within the current standard of care, and are not backed by convincing evidence of safety and efficacy (37). Whether advertising to patients suffering from respiratory diseases or other health problems, such clinics use various persuasive techniques, including patient anecdotes and testimonials by celebrities, to make unproven cell-based products appear safe, effective, and cutting-edge. Sales pitches emphasize narratives of individual empowerment and medical freedom and sometimes contest the role of patient safety and consumer protection regulatory bodies. Some businesses process tissues and cells onsite, whereas other businesses order their cell product from cord blood banks, biobanks, and other suppliers. A growing body of case reports document serious injuries in patients who have been administered unlicensed and unproven stem cell products (38). Clients of such businesses have also suffered significant financial losses with no improvement in health status. Beyond these individual harms, there have also been collective harms, as when patients seek care at clinics that do not conduct research in a careful and systematic manner, underreport adverse events, fail to make meaningful contributions to translational science, and draw individuals away from participating in credible clinical trials.

Responding to the proliferation of such clinics, scientific societies such as the International Society for Cell & Gene Therapy (ISCT), the International Society for Stem Cell Research (ISSCR), Stem Cells Australia, and Canada’s Stem Cell Network have issued guidance to combat misinformation and disinformation and help patients identify misleading advertising claims and distinguish evidence-based cell therapies from unproven cell-based interventions. Businesses offering unproven cell products for lung diseases have proliferated in recent years despite the absence of strong safety and efficacy data for their advertised interventions (39). Respiratory societies, such as the American Thoracic Society and the American Lung Association, and lung patient foundations, such as the Pulmonary Fibrosis Foundation, the Alpha-1 Foundation, and the COPD Foundation have taken a strong stance against such interventions and have issued patient guides, advisories, and guidelines (40). For example, both the Alpha-1 Foundation (https://www.alpha1.org/Buyer-beware-Alphas-urged-to-avoid-companies-advertising-unproven-stem-cell-treatments-that-can-cost-you-a-fortune) and the Pulmonary Fibrosis Foundation have issued statements urging patients to avoid being victimized by clinics selling unproven stem cell interventions (https://www.pulmonaryfibrosis.org/researchers-healthcare-providers/clinical-resources/position-statements/stem-cell-cell-based-therapies-for-pulmonary-fibrosis). Some academic medical centers have offered regenerative medicine consultation services to help patients distinguish between evidence-based stem cell therapies and unproven interventions and make informed decisions (41). Regulatory bodies have also grappled with the spread of clinics selling unproven products on a direct-to-consumer basis. For example, in the United States, the Food and Drug Administration has recently pursued permanent injunctions against two businesses and issued warning letters and untitled letters to clinics and some of their suppliers. The Food and Drug Administration provides a brief overview of the efforts it has made to protect patients from clinics selling unlicensed stem cell interventions (https://www.fda.gov/news-events/press-announcements/statement-stem-cell-clinic-permanent-injunction-and-fdas-ongoing-efforts-protect-patients-risks). The US Federal Trade Commission, state medical boards, and state attorney general offices have also responded to clinicians who have sold stem cell products outside the professional standard of care or have used deceptive sales tactics to influence patients (42).

Rather than pursuing risky and often costly unproven stem cell interventions, some patients can obtain access to investigational stem cell products by participating in peer-reviewed regulated clinical trials. Most countries also have expanded access or compassionate access programs that enable seriously ill persons to obtain access to investigational cell-based products on a nontrial basis (43, 44). “Right-to-try” legislation has emerged in the United States as an alternative to expanded access; however, this route has less oversight, fewer protections, and offers no assurance that patients will receive the desired investigational products (45).

Featuring presenters Drs. Laertis Ikonomou (University at Buffalo), Patricia Zettler (Ohio State University), and Zubin Master (Mayo Clinic), and co-moderators Drs. Sarah Mojarad (University of Southern California) and Leigh Turner (University of California, Irvine), Session IX provided an overview of the direct-to-consumer marketplace for unproven and unlicensed cell-based interventions for respiratory diseases and other illnesses. It also examined stratagems employed regularly by direct-to-consumer businesses and countermeasures for grappling with this troubling and problematic global marketplace. Although the commercial sale of such products shows no signs of abating, there are some encouraging signs of more robust responses by regulatory bodies and efforts to address loopholes, gaps, and areas of ambiguity and confusion in existing regulatory structures. The FDA in particular has helped promote public understanding by explaining the role it plays in supporting the development of safe and efficacious stem cell therapies while alerting patients and consumers to risks associated with the sale and administration of products unsupported by convincing evidence of safety and efficacy (see, e.g., https://www.fda.gov/news-events/fda-voices/advancing-development-safe-and-effective-regenerative-medicine-products and https://www.fda.gov/vaccines-blood-biologics/consumers-biologics/important-patient-and-consumer-information-about-regenerative-medicine-therapies).

## SESSION XI: DIFFERENTIATING AND DELIVERING STEM CELL THERAPEUTICS TO THE LUNG

There are at present no available therapies to halt and then repair the progressive damage that develops with many chronic respiratory diseases. Regenerative therapies for the lung aim to repair or replace damaged tissue through delivery of either therapeutic agents or exogenous stem cells that facilitate lung regeneration. Stem cell (SC)-based therapies hold promise for restoring pulmonary function, but approaches must consider region-specific factors in the lung affecting cell differentiation, function, and signaling. Recent research investigating conditional blastocyst complementation has demonstrated how pluripotent SCs can generate whole functional lobes (46). In these experiments, transgenic mouse lines that are genetically engineered to disrupt core lung development were combined with functionally sufficient pluripotent cells to generate viable chimeric embryos. In these animals, SCs developed a functional pulmonary system due to an available niche and genetic selective pressures.

Session presenter Dr. Vlad Kalinichenko (Cincinnati Children’s Hospital Medical Center) showed that in *Nkx2-1* gene-deleted mouse embryos, which otherwise lack pulmonary tissues entirely, functionally wild-type mouse embryonic stem cells (ESCs) generated pulmonary airways, alveoli, vasculature, and stroma (47). In addition, mouse ESCs that are implanted into a wild-type rat blastocyst can also robustly contribute to pulmonary tissues, such as pulmonary endothelium, and other organs (12), and these interspecies chimeras are a provocative platform for sourcing well-differentiated pulmonary SCs or tissues. Dr. Kalinichenko pointed out that while interspecies chimeras offer an imaginative approach for future regenerative medicines, the possibility of off-target integration into organs such as the brain or reproductive organs must be eliminated before this technology can be translated to clinical applications with pluripotent human cells. However, interspecies chimeras could, one day, be an efficient, ethical source for human pulmonary cells.

Additional future therapies may interact with endogenous cells and their microenvironment to restore pulmonary function and reverse disease pathology. Dr. Jae-Won Shin (University of Illinois at Chicago) presented recent studies from his laboratory demonstrating that therapeutic MSCs may enhance pulmonary regeneration through paracrine immunomodulation and showed that priming MSCs with chemomechanical factors such as tumor necrosis factor alpha (TNF-α) enhanced monocyte trafficking (48). Furthermore, TNF-α gel-coated MSCs accelerated the resolution of fibrosis in bleomycin-injured mouse lungs (49). These results offer a compelling argument for future combination therapies involving cell-based therapies that may help reverse fibrotic remodeling as they help restore functional pulmonary tissues.

The pulmonary extracellular matrix (ECM) is another key factor influencing regenerative potential. Dr. Denis Bölükbaş (Lund University) is stripping back the lungs to their basic ECM components to investigate how the matrix influences pulmonary regeneration. Dr. Bölükbaş explained how future therapies for emphysema may include the delivery of a suitable matrix composed of novel cytocompatible hybrid hydrogels of alginate and decellularized ECM (50). One focus for future experiments in this area is the use of exogenous AT2s as microcarriers of this ECM material.

Much progress has been made in recent years to understand and direct the differentiation and delivery of SCs to the lung. Although further research is needed to advance prospective regenerative medicines for wide-spread use to treat respiratory diseases, there is a clear urgency to drive innovation. Novel regenerative SC therapies offer hope toward reversing established pathologic changes and restoring lung function.

## SESSION XII: LUNG AGING

As evidenced by the ongoing COVID-19 pandemic, the aged population is at increased risk for developing acute and chronic lung diseases. Understanding how organismal and specifically lung aging of different cellular and noncellular components contribute to the development of lung diseases will lead to novel therapeutic approaches.

Several cellular aging hallmarks such as cellular senescence have been described in the lungs of human subjects with IPF (51, 52). Work from Dr. Mauricio Rojas (Ohio State University) showed that naturally or prematurely aged mice [deficient of a DNA repair enzyme (*Ercc1^−^*^/−^)] develop enhanced lung injury or pulmonary fibrosis when exposed to different stimuli such as bleomycin, Herpes virus, or lipopolysaccharide (LPS) (53–55). Parabiosis experiments demonstrated that soluble factors from wild-type mice protected *Ercc1*^−/−^ mice from lung injury. MSCs from old or *Ercc1*^−/−^ mice failed to convey this protection (53, 54). The defect seems to be associated with senescence of bone-marrow derived MSCs. Notably, MSCs from patients with IPF showed signs of cellular senescence (56). Importantly, IPF MSCs increase bleomycin-induced fibrosis and paracrine senescence. Induced apoptosis and clearance of senescent fibroblasts accelerates the resolution of fibrosis in aged mice (57). Senescent cells can be removed by immune cells, however if this mechanism is impaired, senescent cells accumulate. In IPF, natural killer (NK) and natural killer T (NK-T) cells that can potentially clear senescent cells are significantly reduced, especially in the lower lobes of the lung and show drastically altered gene expression profiles in scRNAseq studies (58). In addition, conditioned medium from IPF fibroblasts impairs the cytotoxicity of NK cells and depletion of NK cells leads to persistence of senescent cells in the lung along with an increase in fibrosis in the bleomycin animal model.

Aging also affects vascular endothelial cells (ECs) as presented by Dr. Giovanni Ligresti (Boston University). In contrast to young mice, bleomycin induces nonresolving fibrosis in old mice (57, 59, 60). The aged mice show a vascular phenotype as evidenced by vascular rarefaction (61). Transcriptomic profiling revealed a loss of EC identity in aged mice following bleomycin injury. Multiomic sequencing demonstrated widespread reduction of chromatin accessibility in aged mouse ECs (62). Vascular repair genes are activated in young but not old ECs upon injury. Many of the vascular repair genes that are differentially regulated are predicted to be under the control of the TF ETS related gene (ERG). Loss of ERG in ECs leads to lung inflammation and pulmonary vascular leak under homeostasis and to persistent fibrosis upon bleomycin challenge. Single-cell sequencing revealed ERG as a putative TF involved in general capillary cell (gCAP) homeostasis that is reduced in aging. Single-cell sequence datasets showed a decrease in gCAPs in IPF lungs, which was verified by FACS analysis.

Dr. Renata Jurkowska (Cardiff University) studies epigenetic mechanisms that regulate cellular differentiation in the lung and explores development of novel therapeutic targets for respiratory diseases. Her laboratory has developed a workflow for cell type-resolved transcriptional and epigenetic profiling from cryopreserved human lungs (63). This workflow was used to identify differentially methylated regions (DMRs) associated with chronic obstructive pulmonary disease (COPD) in primary human lung fibroblasts of patients with mild and severe COPD as compared with no COPD donors. Importantly, many of the identified changes were detected in patients with mild COPD, suggesting that epigenetic reprogramming is an early event in COPD pathogenesis. DMRs are enriched in promoters and enhancers, facilitating the discovery of novel master regulator genes of COPD development and/or progression. High throughput knockdown screens confirmed the important role of many of the hits. Notably, several hits showed a differential response in diseased versus donor cells. Looking ahead, the engineering of epigenetic domains for stable gene regulation will be a promising tool to correct disease-specific epigenetic reprogramming.

In summary, defining cell-specific cellular aging phenotypes across the disease spectrum is a promising approach to enhance our understanding of lung diseases. Characterizing epigenetic and upstream regulators of the cross talk between resident structural cells and recruited immune cells will hopefully provide the basis for the development of novel diagnostic, prognostic, and therapeutic approaches.

## SESSION XIII: REGENERATION IN RESPONSE TO VIRAL INFECTION

The COVID-19 pandemic has highlighted the need to understand how the lung responds to and recovers from injury (64, 65). Viral pneumonia is a major cause of severe lung injury manifesting as ARDS (66–68), a common disease with high mortality and no specific therapy (65, 69, 70). Better understanding of lung injury and repair mechanisms might yield new therapeutic approaches that reduce lung injury or promote repair (71). Session XIII highlighted exciting new research toward these goals.

Progress toward enhancing alveolar repair and regeneration has been hampered by insufficient model systems for studying human alveolar epithelial cell disease pathogenesis and generating alveolar epithelial cells for cell-based therapy (18, 72). Dr. Darrell Kotton (Boston University) presented new findings addressing these issues using human AT2-like cells derived from directed differentiation of human iPSCs (73). This population, called iAT2s, demonstrates key features of mature AT2 cells including surfactant production (73, 74). Dr. Kotton’s group found iAT2s are susceptible to SARS-CoV-2 infection, upon which they upregulate proinflammatory and antiviral gene expression (74). Surprisingly, the cathepsin B/L inhibitor E-64d, which blocks SARS-CoV-2 infection in nonalveolar epithelial cell lines, lacks efficacy in iAT2s (74), reinforcing the importance of using relevant cell models for drug screening. Finally, Kotton and colleagues demonstrated syngeneic transplantation of murine iPSC-derived lung epithelial progenitor cells into bleomycin-injured lungs of immunocompetent mice led to apparent engraftment of the cells into lung alveoli, as evident by morphology, protein expression, and gene expression similar to endogenous AT1 cells. Group discussion emphasized the importance of addressing ongoing challenges for the field, including demonstrating function of transplanted cells in the lung and determining lung conditioning regimens that promote transplanted cell engraftment.

Administration of MSCs represents another approach to cell-based therapy for ARDS (75). Most agree that MSCs do not represent stem cells with tissue reconstitution capability, but rather serve as a source of pro-repair mediators upon delivery to damaged tissues (76). In this session, data were presented on an industry sponsored trial of bone marrow-derived MSCs, a standardized MSC product in patients with COVID-19-associated ARDS. Data were promising with clinical improvement, particularly in patients aged younger than 65. The therapeutic use of MSCs and their derived products is moving toward clinical utility; however, it is clear that barriers still remain in achieving significant clinical efficacy.

Chronic lung diseases confer increased risk of severe COVID-19 (77–80), but mechanisms are unclear. Dr. Ben Calvert (University of Southern California) proposed the risk associated with cystic fibrosis (80) and some asthma subgroups (78) relates to the presence of chronic airway neutrophilia. Dr. Calvert found neutrophils associated with lung epithelial damage in fixed lung tissue from patients with COVID-19, suggesting neutrophils play a role in COVID-19 pathogenesis. Presence of neutrophils in culture with airway epithelium increased epithelial secretion of proinflammatory cytokines in response SARS-CoV-2, and airway epithelium exposed to neutrophils or cytokines had loss of transepithelial resistance and increased SARS-CoV-2 replication. Taking these findings together, Calvert and colleagues conclude that neutrophils augmented the airway epithelial proinflammatory response to SARS-CoV-2 and suggest that neutrophil-epithelial interactions promote viral replication and virus-induced airway epithelial barrier loss. Reports by others (81) support Dr. Calvert’s findings by demonstrating lung epithelial cells from patients with chronic lung diseases have unique expression of genes related to viral replication and immunity. Clearly, the role of chronic neutrophilia in COVID-19 pathogenesis warrants further study.

## SETTING PRIORITIES AND RECOMMENDATIONS REGARDING FUNDING FUTURE RESEARCH

The broad field of lung regenerative medicine continues to evolve at an accelerating pace. The NIH, nonprofit respiratory disease foundations, and other sources of scientific and funding support remain positive and will be important for continued development. As in past conference reports, a series of scientific and funding recommendations coming out of discussions at the conference and post-conference surveys are presented in Table 1. These recommendations continue to evolve and reflect a growing interest in application of bioengineering approaches and technologies, including recent advances in imaging technologies, to the study of lung biology and diseases. Continuing a new feature initiated during the 2019 conference, a series of survey questions was conducted in the final session addressing key theme areas of the conference including advances in biotechnology and bioengineering, cells utilized in different regenerative approaches, cell-based therapies, and use of 3-D model systems. An audience response system using individual voting remotes was used for immediate anonymous responses from participants of the conference (https://doi.org/10.6084/m9.figshare.19406570) to guide discussion and crafting of the overall recommendations.

**Table 1.** Conference summary, recommendations, and focus areas

*Basic Science: Analysis and Visualization of Endogenous Lung Stem Cells*
Continue progress in applying a systems-level approach to discover interactions through mining of single-cell omics data to identify fundamental cell signaling pathways and networks in homeostasis and disease.
Identify cell surface markers that characterize lung cell populations for use in visualization and sorting techniques to identify functional subpopulations with potential for use in regenerative approaches.
Establish techniques for light-sheet microscopy, including live imaging and imaging in human tissue. The limited number of techniques that can be used to image lung regeneration at high-spatiotemporal resolution was reiterated throughout the workshop. Current techniques are limited by difficulties in penetrating thick tissue samples, long imaging acquisition times, and phototoxicity
*Basic Science: Induced Pluripotent Stem Cells and Disease Modeling*
Refine and functionally validate protocols to derive lung cells from pluripotent stem cells (iPSCs and ESCs). Critical comparisons to primary lung cells.
Continue to develop high-throughput cellular models from pluripotent sources for screening of novel therapeutics.
Comprehensively evaluate the effect of environmental influences, including mechanical forces, extracellular matrix, inflammation, and infection on development of lung tissue from stem and progenitor cells.
*Basic Science: Understanding of the Impact of Stem Cell: Niche Interactions*
Continue to elucidate how endogenous lung stem/progenitor cells are regulated in normal development and during tissue homeostasis.
Analyse epigenetic modulation of lung stem cells and how cellular microenvironments change this.
Understand how autologous iPSC-derived lung lineages will behave in the diseased/aged microenvironment in vivo.
*Basic Science: Bioengineering and the Lung*
Continue to explore lung tissue bioengineering approaches such as artificial matrices, 3-D culture systems (e.g., extracellular matrix environments for organoid culture), 3-D bioprinting and other novel approaches for generating lung ex vivo and in vivo from stem cells, including systems that facilitate vascular development
Evaluate effect of environmental influences, including oxygen tension, and mechanical forces, including stretch and compression pressure, on development of lung tissue from stem and progenitor cells.
Define the consensus endpoints for functional evaluation and validation of engineered lung tissue.
*Translational Science: Cell Therapy—Delivery of Stem Cells to the Lung*
Invest in developing larger animal models, such as the ferret and the pig, that have lung structure and function more akin to that of humans, as preclinical models to evaluate cellular therapy.
Integrate lung stem cell science into multidisciplinary teams (e.g., with clinical, surgical, regulatory, informatics input, etc.).
Act as a community to prevent the proliferation of dubious therapies based on “stem cell” branding while supporting the development of evidence-based stem cell interventions.

Many of the recommendations from our previous conferences remain valid with priorities for research focus and funding (82). The current table comprises new recommendations arising specifically from the 2021 conference. 3-D, three-dimensional; ESCs, embryonic stem cells; iPSCs, induced pluripotent stem cells; MIT, Massachusettes Institute of Technology; PCLS, precision cut lung slices; RFA, request for applications.

**Figure 1.**
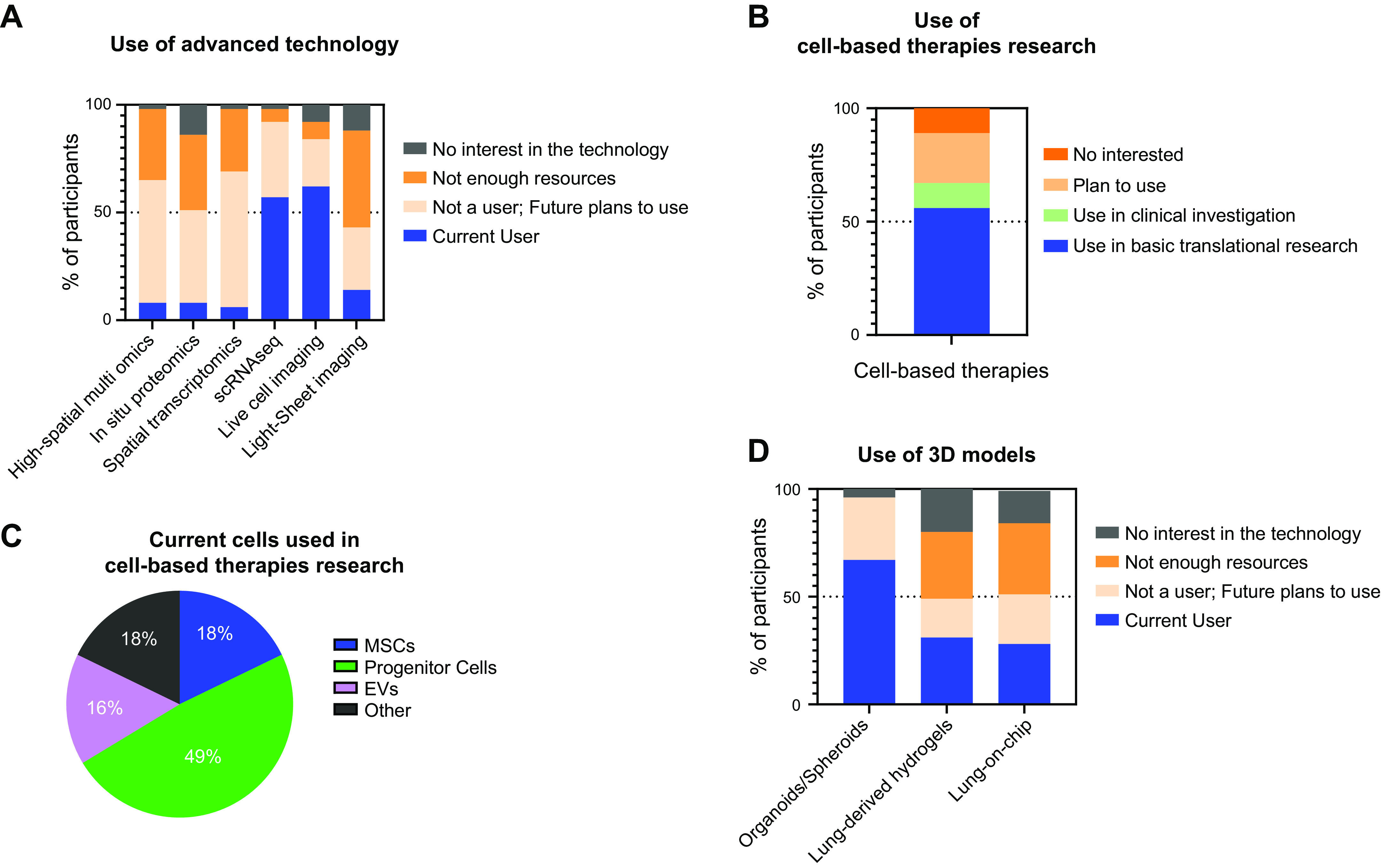
Summarized survey data for the open discussion of conference content. *A*: use of advanced technologies such as single-cell RNAseq (scRNAseq) and live imaging. *B*: investigating cell-based therapies. *C*: types of cells being used in cell-based therapy research. *D*: use of three-dimensional models in research. EVs, extracellular vesicles; MSC, mesenchymal stromal cell.

In brief, the survey indicated large and increasing numbers of individuals and research groups utilizing technologies such as scRNAseq and live imaging as well as keen interest in other cutting-edge technologies such as light sheet imaging (Fig. 1*A*). Not surprisingly, lack of access or limited resources to use more advanced technologies remains a limiting factor. Robust discussion was held about greater access to core facilities or centers with advanced technologies and how to best fund a wider range of potential users. Sixty-seven percent of respondents indicated that they are currently investigating cell-based therapies, 56% in basic/translational research and 11% in clinical investigation (Fig. 1*B*). As in the 2019 survey, the majority, 49%, are investigating endogenous progenitor cells with 18% investigating in MSCs, 16% investigating extracellular vesicles (EVs), and 18% investigating a range of other cells including iPSCs and endothelial progenitor cells (EPCs). The survey also highlighted the support for a broad range of approaches (Fig. 1*C*). Notably 97% of respondents indicated current or future planned activities with organoid culture approaches but only 31% and 28% of respondents indicated activity or interest in either hydrogel or lung-on-a-chip approaches (Fig. 1*D*). Other survey questions and responses including incorporation of bioethics training are available at https://doi.org/10.6084/m9.figshare.19406570.

## CONCLUSIONS

Since the last conference in 2019, substantial advances in the development of new technologies, increasing utilization of scRNAseq data, and the further development of more physiologically relevent 3-D cellular models have driven considerable progress in the field moving closer to the development of potential therapeutic strategies for lung repair after injury or in end-stage lung disease. Despite these developments, there are still considerable deficits in our understanding of how transcriptomic changes in different endogenous and derived stem and progenitor cell populations correlate to function at the tissue and organ level, how to describe the stem cell niche, and how changes in this niche lead to functional alterations in these cells. The pursuit of understanding the function of human lung across the spectrum from a subcellular to organ-level to improve success of therapeutic translation of our findings will be increasingly dependent on collaborative and interdisciplinary research teams. This conference continues to bring together leaders across such multidisciplinary areas of active research from basic biologists, bioengineers, and bioinformaticians to ethicists, business leaders, and policy makers and retains a focus on the promotion of innovation among junior scientists across the world. The recommendations in Table 1 compiled from active discussion throughout the conference should provide a platform for stimulating active new research that will progress the field toward its goals for restoration of a functional lung in patients living with acute and chronic lung disease.

## GRANTS

This work was supported by National Heart, Lung, and Blood Institute Grants R13HL149436 and R13HL160043-01.

## DISCLOSURES

A.L.R. and D.J.W. received funding from National Heart, Lung, and Blood Institute R13 Conference Grant to prepare the conference. R.E.H. is a Wellcome Trust Sir Henry Wellcome Fellow (WT209199/Z/17/Z). None of the other authors have any conflicts of interest, financial or otherwise, to disclose.

## AUTHOR CONTRIBUTIONS

L.I., M.M., R.D., E.L.H., R.E.H., Z.B., J.-A.P., S.S., J.K.B., L.T., S.M.M., J.E.M., T.L., M.L., V.J.T., J.L.H., A.E.V., E.T.H., D.J.W., and A.L.R. drafted manuscript; L.I., M.M., R.D., E.L.H., R.E.H., Z.B., J.-A.P., S.S., J.K.B., L.T., S.M.M., J.E.M., T.L., M.L., V.J.T., J.L.H., A.E.V., E.T.H., D.J.W., and A.L.R., edited and revised manuscript; L.I., M.M., R.D., E.L.H., R.E.H., Z.B., J.-A.P., S.S., J.K.B., L.T., S.M.M., J.E.M., T.L., M.L., V.J.T., J.L.H., A.E.V., E.T.H., D.J.W., and A.L.R. approved final version of manuscript.
